# Psychological Stress and the Cutaneous Immune Response: Roles of the HPA Axis and the Sympathetic Nervous System in Atopic Dermatitis and Psoriasis

**DOI:** 10.1155/2012/403908

**Published:** 2012-08-30

**Authors:** Jessica M. F. Hall, desAnges Cruser, Alan Podawiltz, Diana I. Mummert, Harlan Jones, Mark E. Mummert

**Affiliations:** ^1^Department of Molecular Biology and Immunology, University of North Texas Health Science Center, Fort Worth, TX 76107, USA; ^2^Department of Medical Education, University of North Texas Health Science Center, Fort Worth, TX 76107, USA; ^3^Department of Psychiatry and Behavioral Health, University of North Texas Health Science Center, Fort Worth, TX 76107, USA; ^4^Department of Dermatology, University of Texas Southwestern Medical Center, Dallas, TX 75390, USA

## Abstract

Psychological stress, an evolutionary adaptation to the fight-or-flight response, triggers a number of physiological responses that can be deleterious under some circumstances. Stress signals activate the hypothalamus-pituitary-adrenal (HPA) axis and the sympathetic nervous system. Elements derived from those systems (e.g., cortisol, catecholamines and neuropeptides) can impact the immune system and possible disease states. Skin provides a first line of defense against many environmental insults. A number of investigations have indicated that the skin is especially sensitive to psychological stress, and experimental evidence shows that the cutaneous innate and adaptive immune systems are affected by stressors. For example, psychological stress has been shown to reduce recovery time of the stratum corneum barrier after its removal (innate immunity) and alters antigen presentation by epidermal Langerhans cells (adaptive immunity). Moreover, psychological stress may trigger or exacerbate immune mediated dermatological disorders. Understanding how the activity of the psyche-nervous -immune system axis impinges on skin diseases may facilitate coordinated treatment strategies between dermatologists and psychiatrists. Herein, we will review the roles of the HPA axis and the sympathetic nervous system on the cutaneous immune response. We will selectively highlight how the interplay between psychological stress and the immune system affects atopic dermatitis and psoriasis.

## 1. Introduction

Psychological stress can trigger the activation of numerous physiological responses, including the endocrine, nervous, and immune systems [1–7]. Nearly 100 years ago, Cannon hypothesized that the release of substances (adrenalin, epinephrine, etc.) by the adrenal medulla during “pain and the major emotions” (fear, rage, and asphyxia) was an evolutionary adaptation for survival [8]. For example, an encounter with a predator induces an acute psychological stress which in turn activates the release of substances from the adrenal medulla. Substances released by the adrenal medulla induce profound physiological changes (increased circulation to the lungs, heart and limbs; increased cardiac vigor and increased sugar content in the blood; cessation of the activities of the alimentary canal) that endow the intended prey to flee or to fight. However, the connotation of emotional distress as an adaptation for survival has dramatically changed for most modern humans. Today, for example, there may be psychological stress due to divorce or unemployment, with the peripheral physiological responses associated with stress being unwanted.

 The concept that psychological stress impacts the health of an individual has long been postulated. Accumulating experimental evidence is beginning to delineate how stress can induce or exasperate disease processes. A comprehensive understanding of the mechanisms whereby psychological stress contributes to disease processes may deepen our understanding of the mind-body connection and may provide novel approaches to patient treatment.

 The skin constitutes the largest bodily organ and is bombarded daily with environmental insults including infectious and toxic agents, allergens, ultraviolet light, and mechanical damage. Therefore, the skin is equipped with innate and adaptive properties to respond to the myriad of environmental factors encountered. In addition to environmental factors, skin also appears especially responsive to psychological stressors. Indeed, a number of psychodermatologic disorders associated with stress have been reported, including (1) psoriasis, (2) atopic dermatitis, (3) pruritus, (4) alopecia areata, (5) lichen planus, and (6) rosacea [9]. A plausible interprofessional arena between dermatology and psychiatry is elucidated by studies on outpatients in dermatology clinics showing psychiatric morbidity [10, 11]. In fact, cooccurring psychiatric disorders in patients with skin disorders show a prevalence of around 30% [12]. The purpose of this paper is to review the impact of psychological stress on the cutaneous immune response and highlight the potential role of psychological stress in two skin diseases commonly encountered in the clinic: atopic dermatitis and psoriasis.

## 2. Skin and the Neuroendocrine System

**Figure 1:**
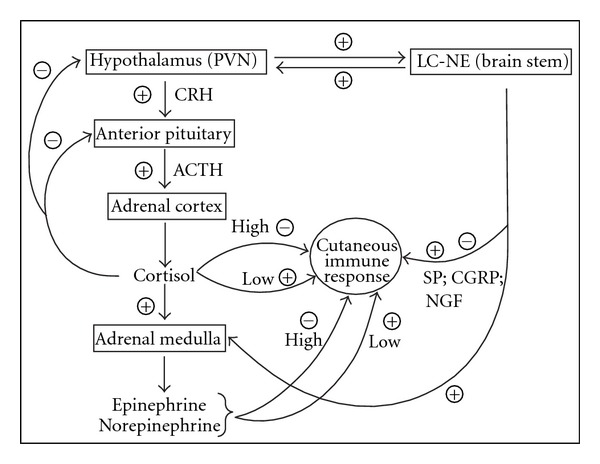
A schematic representation of the hypothalamic-pituitary-adrenal (HPA) axis and sympathetic nervous interaction with the cutaneous immune system. Stress signals induce release of hormones, including corticotropin-releasing hormone (CRH) from the paraventricular nucleus (PVN) of the hypothalamus. CRH induces adrenocorticotropic hormone (ACTH) release from the anterior pituitary [20]. In turn, ACTH regulates glucocorticoid secretion from the adrenal cortex [22]. Cortisol has several functions including negative feedback of the hypothalamus and anterior pituitary and induces epinephrine and norepinephrine from the adrenal medulla [23]. Glucocorticoids, such as cortisol, as well as epinephrine and norepinephrine may enhance cutaneous immune responses at low concentrations and suppress immune responses at high concentrations [5, 28]. Stress signals also stimulate the locus coeruleus (LC) norepinephrine cells (NE) of the sympathetic nervous system [18]. Neuropeptide products of the sympathetic response (substance P (SP), calcitonin gene-related peptide (CGRP), and cutaneous nerve growth factor (NGF)) have been shown to be proinflammatory and anti-inflammatory dependent on the immune cell type [29–34]. There also exists a positive, reverberatory feedback loop between the HPA axis and LC-NE [18, 21]. Results show that HPA and sympathetic stress responses both modify the cutaneous immune response.

 The central hypothalamic-pituitary-adrenal (HPA) axis is activated following stress signals such as 5-hydroxytryptamine [13, 14], acetylcholine [15], and inflammatory cytokines [16, 17]. Stress signals also activate the locus coeruleus (LC) of the brain stem eliciting a sympathetic nervous system response. There exists a positive, reverberatory feedback loop between these two major systems [18]. When the HPA axis is activated, stress hormones are released including corticotropin-releasing hormone (CRH) and arginine vasopressin [19] from the hypothalamus, which induces adrenocorticotropic hormone (ACTH) release from the anterior pituitary [20]. CRH also activates the LC-noradrenergic pathways resulting in norepinephrine secretion by the peripheral sympathetic nervous system and norepinephrine and epinephrine secretion from the adrenal medulla [21]. ACTH regulates secretion of glucocorticoids including cortisol from the adrenal gland [22]. Cortisol negatively regulates CRH production in a feedback loop mechanism [23]. Norepinephrine is a major neurotransmitter released by sympathetic fibers to innervated tissues, including the skin [24–26]. Activation of the sympathetic nervous system also leads to increased production of other factors including catecholamines [27]. A highly schematic overview of the central HPA axis and locus coeruleus/norepinephrine (LC-NE) sympathetic response to stress signals including the downstream effects on the cutaneous immune response is shown in Figure 1.

Investigations have shown that human skin expresses CRH as well as CRH receptors (CRH-R). The CRH-R1*α* isoform is the predominant CRH receptor in skin and is expressed in all major cell populations of epidermis, dermis, and subcutis. By contrast, CRH-R2 is expressed predominately in hair follicles, sebaceous and eccrine glands, muscle and blood vessels [35]. CRH protein is also present in murine skin although CRH mRNA has not been detected [35]. However, both mRNA and protein products for CRH-R1 and 2 have been detected in murine skin [36]. In addition to CRH, human skin also expresses urocortin I [37] and urocortin II mRNA [35]. CRH-R1 binds to urocortin I, but not to urocortin II; while CRH-R2 binds to urocortin II, but not urocortin I [38, 39] leading us to belief that the skin has a depth of responsiveness and interaction to the environment that is little understood. Finally, skin produces the precursor protein, proopiomelanocortin protein (POMC) and POMC derived peptides that give rise to ACTH and other polypeptide products [40, 41].

Ito et al. have shown that human hair follicles can synthesize cortisol and that cortisol synthesis is regulated by endogenous feedback controls [42]. Thus, the skin apparently has a peripheral equivalent of the HPA axis that is fully functional. The peripheral skin HPA axis may coordinate or fine tune peripheral stress responses with the central HPA axis. In addition to expressing components of the HPA axis, skin also produces a number of other neuroendocrine signals including prolactin [43–45], melatonin [46], and catecholamines [47, 48].

 In addition to the HPA axis, the skin is highly innervated with sensory nerves that produce neurotrophins and neuropeptides. Sensory nerves derive from the dorsal root ganglion in the skin and C-fibers form the cutaneous sensory nervous system. Psychological stress leads to increased concentrations of cutaneous nerve growth factor (NGF) [29]. NGF has a number of biological activities including (1) axon sprouting of peptidergic and sympathetic neurons, (2) promoting cross-talk between neural cells, glia, and immune cells, and (3) facilitating monocyte/macrophage migration through vascular endothelium [30]. NGF upregulates SP+ nerve fibers in the dermis of stressed mice. Calcitonin gene-related peptide (CGRP), a potent vasodilator, is also upregulated in response to NGF [29]. SP and CGRP have different distributions within the skin with SP nerve fibers detected in the dermis and subcutis and CGRP nerve fibers are in the epidermis around the distal hair follicle and the arrector pili muscle [31].

## 3. Impact of Psychological Stress on Innate and Adaptive Immunity in the Skin

 The innate immune response consists of elements that contribute to the immediate and generic defense of the skin; immunological memory does not develop. By contrast, the adaptive immune response requires time for the development of a specific defense and can create immunological memory. Psychological stress has been shown to impact both innate and adaptive immune responses.

### 3.1. Innate Immune Responses to Stress

The stratum corneum is terminally differentiated epidermis that forms the outer most layer of the skin. The corneocytes forming the stratum corneum arise from the underlying keratinocytes although unlike their predecessors, corneocytes, lack nuclei and most cell organelles. The intercorneocyte spaces contain high concentrations of nonpolar lipids contributing to the water impermeability of the stratum corneum. The stratum corneum plays an integral role in maintaining tissue hydration, and its mechanical or chemical disruption results in transepidermal water loss. In addition to its role in hydration, the stratum corneum is normally sloughed off, potentially removing skin microorganisms such as potential pathogens. Finally, the stratum corneum contains melanocyte-derived melanin that protects the skin from ultraviolet radiation [49]. Denda et al. evaluated the impact of immobilization stress and crowding stress on the barrier function of the stratum corneum as measured by barrier recovery after its removal by tape stripping or sodium dodecyl sulfate treatment in rats. In that study, immobilization induced stress and crowding stress both significantly delayed barrier recovery for up to 7 days in both male and female mice. Interestingly, the tranquillizers diazepam and chlorpromazine resulted in an increased rate of barrier recovery. Thus, pharmacological reduction of psychological stress promoted stratum corneum formation [50].

Garg et al. evaluated the impact of psychological stress on barrier recovery in humans [51]. Individuals with high levels of perceived psychological stress had significantly delayed barrier recovery rates as compared with those reporting low perceived stress levels. These investigators concluded that stress-induced changes in epidermal function may serve as precipitators of dermatoses. Using SKH-1 mice and stress induced by continuous light and radio noise, Choi et al. found that impaired stratum corneum barrier function could be linked to decreased synthesis of epidermal lipids [52]. Choi et al. hypothesized that increased concentrations of glucocorticoids could result in the epidermal abnormalities observed during psychological stress, including the delay in the stratum corneum barrier recovery [53]. Subsequent treatment of psychologically stressed mice with RU-486 (a glucocorticoid receptor antagonist) or antalarmin (a CRH antagonist that blocks increased glucocorticoid production) returned stratum corneum recovery to normal rates. These results highlight the importance of glucocorticoids induced during psychological stress and stratum corneum homeostasis.

 Skin also synthesizes and secretes antimicrobial peptides encapsulated in lamellar bodies. Aberg et al. evaluated the impact of cutaneous *Streptococcus pyogenes* infections on psychologically stressed mice [54]. Animals stressed by continuous light and radio noise downregulated the antimicrobial peptides (cathelin-related peptide and *β*-defensin) and developed correspondingly more severe *S. pyogenes* cutaneous infections as compared with nonstressed control mice. Pharmacological blockade of CRH or glucocorticoid production returned antimicrobial peptides to normal levels and reduced the infection severity. Thus, psychological stress appears to be directly linked to the innate immunity conferred by antimicrobial peptides via the central or peripheral HPA axis.

 Mast cells are found throughout connective tissues, including the dermis [55–57]. ACTH and CRH activate mast cells, and human mast cells express CRH receptors [58]. Recent work by Asadi et al. has shown that SP can induce the expression of functional CRH receptor-1 in human mast cells [32]. Acute psychological stress is linked with mast cell activation and the release of IL-6. The finding that serum levels of IL-6 are abrogated in mast cell-deficient mice following restraint stress as compared with their wildtype counterparts underscores the importance of mast cells in the production of systemic IL-6 [59]. Importantly, IL-6 can cross the blood/brain barrier [60] and activate the HPA axis [61]. IL-6 can also induce immune reactions including lymphocyte activation [62, 63] and increased antibody production via CD4^+^ T-cell help [64]. Systemic effects of IL-6 include induction of fever [65] and acute phase protein production [66, 67].

Mast cells also play a role in neurogenic inflammation. Singh et al. reported that restraint-induced stress resulted in significantly enhanced degranulation of mast cells in mice as compared with their nonstressed counterparts. Pretreatment of mice before stress with CRH antiserum, the neurotensin receptor antagonist SR48692 and capsaicin to deplete sensory neurons were all found to inhibit mast cell degranulation. These results suggested a role for neurogenic inflammation in psychological stress that is in addition to the HPA axis [68]. In fact, a number of investigators have shown that psychological stress activates the mast cell/nerve fiber interface leading to neurogenic inflammation [69, 70]. Shimoda et al. reported that administration of an antipsychotic drug (chlorpromazine) and anxiolytic reagents (tandospirone and CRA1000) significantly reduced degranulation of dermal mast cells in mice stressed by electric foot shock [71]. These results may suggest that antipsychotic and anxiolytic agents may be effective treatments for stress-aggravated inflammatory skin diseases by inhibition of mast-cell degranulation [71].

### 3.2. Adaptive Immune Responses to Stress

The adaptive immune response requires the interaction of antigen-presenting cells (i.e., dendritic cell) with antigen-specific lymphocytes (i.e., T cells). Activation of lymphocytes requires their complex interplay with antigen presenting cells and co-stimulatory molecules on the surfaces of both cell types as well as the production of cytokines.

Dhabhar and Mcewen investigated the impact of acute stress on contact hypersensitivity (CHS) reactions in rats using stress induced by a 2-hour confinement in a plexiglass box [4]. Briefly, animals were sensitized using 2,4-dinitrofluorobenzene (DNFB), stressed on day 5 following sensitization and challenged on the pinna of the ear on day 6 with ear swelling used as the read-out after the DNFB challenge. Acute stress markedly increased the ear swelling response in stressed rats as compared with the control animals. Elimination of glucocorticoid and epinephrine by adrenalectomy eliminated the stress-induced enhancement, underscoring the importance of these hormones for immunomodulation. Moreover, administration of corticosterone or epinephrine at low doses enhanced stress-induced ear swelling suggesting that these hormones play a role in immunoenhancement. On the other hand, high doses of corticosterone or epinephrine had the opposite effect, that is, ear swelling was reduced. Therefore, the outcome of corticosterone and epinephrine depends on their concentrations. Using a different contact sensitizing reagent (trinitrochlorobenzene) and isolation stress, Nakano also found that stress enhanced the cutaneous immune response as evaluated by ear swelling [72]. However, stress alone did not enhance the ear swelling response of mice treated with the contact irritant, sodium dodecyl sulfate. These results suggested that elements of the adaptive immune response were required for acute stress-induced immune enhancement, as irritants do not develop immunological memory.

In contrast to the results described above, Flint et al. showed that restraint stress prior to DNFB sensitization resulted in suppression of the immune response [73]. Thus, experimental studies have provided seemingly contradictory results. It is, therefore, tempting to speculate that the nature of the sensitizing agent, the dose of the contact sensitizing agent and the timing of the stressor are all variables that are important for the ensuing immune response.

Different strains of mice may have different skin sensitivities to psychological stressors. Flint et al. reported that C57BL/6 mice had blunted ear swelling responses to restraint stress as compared with BALB/c mice [74]. Importantly, ear swelling responses in stressed C57BL/6 strain could not be enhanced even after exogenous corticosterone. The nature of the stressor may also impact the magnitude of the CHS response in animals. Bowers et al. compared CHS responses in mice acutely or chronically stressed by restraint, forced swim, isolation, handling, and low temperature [7]. Restraint stress and forced swim stress resulted in the most dramatic increase in the CHS response as assessed by the ear swelling assay. Taken all together, experimental studies have shown a correlation between acute stress and contact hypersensitivity responses in rodents. However, further investigations are required to delineate conditions under which acute stress suppresses immune responses and under which conditions acute stress enhances immune responses. Findings that the outcomes of acute psychological stress are related to mouse strain also suggest that genetic background may impact the interplay between stress and skin inflammation.

The impact of chronic stress on skin immune responses has also been investigated. Chronic stress has been reported to lead to immunosuppression in a number of systems, including skin graft rejection [75]. In a model of chronic restraint-induced stress, the CHS response was markedly suppressed [3]. By contrast, other studies using chronic restraint-induced stress resulted in enhanced CHS responses [7]. The thyroid axis may also modulate immune responses during chronic stress [76].

Among the factors that may account for psychological stress-induced changes in the adaptive immune response are changes in the numbers, proportions, and distributions of immune cells. Previous studies found that psychological stress markedly decreased the percentages of leukocytes in the blood [1, 2]. Interestingly, administration of corticosterone to adrenalectomized mice closely mirrored the decrease in blood leukocytes observed in stressed animals [2].

In addition to differences in cell numbers and distributions, psychological stress may modulate the activities of immunological cells. For example, stress has been shown to impair lymphocyte function [77]. Stress has also been shown to decrease the density of Langerhans cells in the epidermis in both mice and humans [78, 79]. Langerhans cells are epidermal members of the dendritic cell family of antigen-presenting cells. Conventionally, Langerhans cells have been considered pivotal for the generation of adaptive immunity although current studies suggest that their immunological activities may be considerably more complex (reviewed in [80]). A number of stress related molecules have been shown to impact Langerhans cells and dendritic cells. Hoetzenecker et al. have shown that corticosteroids induce the apoptosis of Langerhans cells and impair their expression of costimulatory molecules [81]. Studies in vitro have shown that epinephrine inhibits antigen presentation in epidermal cell preparations as well as in purified Langerhans cells [82]. Glucocorticoids inhibit dendritic cell production of IL-12 [83, 84]; and IL-12 suppression may skew the T_H_1/T_H_2 balance toward T_H_2 and thus impact the nature of the immune response [85]. Importantly, blockade of *β*2-AR with the antagonist ICI188, 551 impaired the migration of Langerhans cells to the lymph nodes and blunted the subsequent CHS response when mice were sensitized with the fluorescein isothiocyanate contact sensitizing reagent [82]. In contrast to impaired dendritic cell activities, other stress-related molecules appear to enhance dendritic cell functions. For example, Yanagawa et al. showed that noradrenaline enhanced phosphatidylinositol 3-kinase-induced antigen endocytosis by dendritic cells in vitro [86]. 

Neuropeptides can also impact the biological activities of antigen-presenting cells. Hosoi et al. showed that CGRP impinged on Langerhans cells in the epidermis and CGRP was directly detected on the surfaces of some Langerhans cells. Moreover, CGRP was found to inhibit the ability of Langerhans cells to present antigen in vitro [33]. Recently, Ding et al. showed that treatment of Langerhans cells with CGRP decreased antigen presentation to a T_H_1 T-cell clone but increased antigen presentation to a T_H_2 T-cell clone. Those researchers suggested that exposure of Langerhans cells to nerve-derived CGRP may polarize the immune response to a T_H_2 type of immunity [87]. Other neuropeptides may also modulate the ability of Langerhans cells to effectively present antigen. Staniek et al. found that SP can bind to human Langerhans cells and impair T-cell proliferative responses in the mixed epidermal-cell lymphocyte reaction. Based on those results the investigators concluded that SP can impair antigen presentation [34].

In summary, psychological stressors and stress-related molecules (e.g., epinephrine, glucocorticoids, and noradrenaline) have been shown to impact various cell behaviors, costimulatory molecule expression and cytokine profiles of immune cells in skin adaptive immune responses, including dendritic cells and lymphocyte immune cell subsets.

## 4. Psychological Stress and Human Skin Diseases

 A number of skin diseases may be preceded or exacerbated by psychological stress. In the following section, we review what is known about the impact of psychological stress on atopic dermatitis and psoriasis. Our focus is on these two skin diseases because they are relatively common skin disorders.

### 4.1. Atopic Dermatitis

 Atopic dermatitis is a chronic inflammatory skin disorder characterized by eczematous lesions and pruritus. It is a common disorder affecting 6% of the population in the USA [88]. Atopic dermatitis may be the result of genetic predisposition and environmental conditions, and no single etiologic agent is known. Analyses of sequential patch-testing skin biopsies have suggested that atopic dermatitis has a biphasic T_H_1/T_H_2 T-cell response. Acute inflammation is primarily T_H_2 with a shift toward T_H_1 chronification [89, 90]. Psychological stress is known to aggravate atopic dermatitis and a psychological profile that includes anxiety, depression, and excitability has been linked to this disease [91]. Traumatic events, including natural disasters, may increase psychological stress in the population at large, exasperating the incidence of atopic dermatitis symptoms. For example, after the Great Hanshin earthquake in January 1995, subjective distress was found to be the root cause for the enhanced symptoms of atopic dermatitis in the populations of the affected geographic areas [92].

Buske-Kirschbaum et al. analyzed leukocyte subsets, serum IgE levels, and cytokine concentrations in atopic dermatitis patients and nonatopic controls stressed in front of an audience using the Trier Social Stress Test (TSST) [93]. Both groups showed significant elevations in the numbers of serum lymphocytes, monocytes, neutrophils, and basophils with no differences between the two groups. However, eosinophil numbers were significantly higher in atopic dermatitis patients as compared with nonatopic controls. Similarly, IgE levels were significantly greater in the atopic dermatitis patients than their nonatopic counterparts. In both groups, TSST resulted in increased concentrations of IFN*γ* and a reduction in IL-4 concentrations with no significant differences between the two groups. These studies showed that immunological similarities and differences exist between atopic dermatitis patients and nonatopic individuals subjected to psychological stressors. 

Interestingly, patients with atopic dermatitis have been shown to have reduced production of cortisol and ACTH due to experimental TSST stressors as compared with nonatopic controls. By contrast, catecholamine levels were significantly higher in atopic patients as compared with nonatopic controls. Thus, atopic dermatitis patients have blunted HPA axis reactivity as assessed by cortisol and ACTH measurements, but an overactive sympathetic adrenomedullary system as suggested by the high concentrations of catecholamine [94]. Both the HPA axis and the SAM system suppress T_H_1 activity potentially via IL-12, thus skewing the T_H_1/T_H_2 balance toward T_H_2. Thus, flares in atopic dermatitis following psychological stress may reflect T_H_2 skewing to acute disease symptoms.

 Concentrations of NGF and SP are elevated in in the sera of atopic dermatitis patients. Moreover, NGF and SP concentrations have been positively correlated with disease severity [95, 96]. Recently, Lonne-Rahm et al. compared skin biopsies from patients with atopic dermatitis and chronically stressed atopic dermatitis patients [97]. Cortisol concentrations were used to define which patients were psychologically stressed. In both groups, the CD3^+^ cell infiltrates expressed the 5-hydroxytryptamine 2A receptor and the serotonin transporter protein. Furthermore, the numbers of mast cells were significantly greater in the skin lesions as compared with uninvolved skin. Likewise, nerve fibers were found in the epidermis and papillary dermis of involved skin as compared to uninvolved skin. In contrast, the number of SP and CGRP positive nerve fibers was not significantly different between involved and noninvolved skin. Nonetheless, chronic stress was correlated with greater numbers of 5-hydroxytryptamine 2A receptor positive cells in the papillary dermis of involved skin. These results showed that atopic dermatitis results in differences in skin innervation and modulation of the serotonin system that also occurs in atopic dermatitis patients during chronic stress.

In summary, atopic dermatitis is a dermatological disorder that is characterized initially as an acute T_H_2-mediated disease that becomes T_H_1 polarized with chronicity. Atopic dermatitis seems to worsen in patients that are psychologically stressed, and adult atopic dermatitis patients have a constellation of psychological conditions that may place them at risk for this dermatitis. Finally, it has been reported that atopic dermatitis patients have a blunted HPA response and an overactive sympathetic adrenomedullary system that may exacerbate disease.

### 4.2. Psoriasis

Approximately 2% of the population in the USA is diagnosed with psoriasis [98]. Most newly diagnosed psoriasis patients are under the age of 30. Psoriatic arthritis, which is potentially debilitating, develops in 10–40% of psoriatics [99, 100].

 Psoriasis is a multifactorial disease shaped by genetics and environmental factors that include psychological stress [101]. Currently, it is believed that T cells play a significant role in disease pathogenesis, particularly T cells expressing IL-17. Kryczek et al. have proposed that activated T_H_1 cells are recruited into the skin and secrete IFN*γ*. In turn, the IFN*γ* induces local antigen-presenting cells to secrete IL-1 and IL-23 that promote the expansion and survival of IL-17 expressing CD4^+^ and CD8^+^ T cells. Trafficking of IL-17 expressing CD8^+^ T cells into the epidermis then promotes epidermal hyperplasia [102]. The T-cell-activating antigen(s) remain unknown.

 Psychological stressors have been reported to precede the onset of psoriasis in 44% of patients and to initiate recurrent skin flares in 88% of psoriatics [103, 104]. Buske-Kirschbaum et al. have reported psoriatics exposed to the TSST stressor had greater numbers of CD4^+^ T cells and monocytes in their blood as compared with a nonpsoriatic control group. On the other hand, numbers of CD3^+^/CD25^+^ T cells were decreased in psoriatics as compared with nonpsoriatic controls. Psychological stress increased the numbers of CD3^+^, CD8^+^, CD16^+^/CD56^+^ (i.e., NK cells), and CD3^+^/HLA-DR leukocytes in the blood, although the differences between psoriatics and nonpsoriatics were insignificant [105]. Schmid-Ott et al. evaluated circulating levels of T cells and NK cells in psoriatics and nonpsoriatic following experimental psychological stress [106]. In contrast to results obtained by Buske-Kirschbaum et al., levels of CD3^+^ T cells increased significantly only in the blood of psoriatics following psychological stress. Importantly, increased T-cell counts were due to increased numbers of CD8^+^ and CD3^+^CLA^+^ T cells (CLA, cutaneous lymphocyte-associated antigens). Similarly, CLA^+^ NK cells were increased significantly only in the circulation of psoriatics following psychological stress. Importantly, the CLA molecule is required for the trafficking of the cells to skin.

 Interestingly, psoriasis patients who reported stress-exacerbated flares were found to have decreased levels of cortisol and epinephrine [107]. Thus, similar to atopic dermatitis patients, it would appear that the response of the HPA axis is blunted in psoriatics sensitive to psychological stressors. By contrast, Karanikas et al. recently reported that HPA axis reactivity was not correlated with psychopathological and immune parameters in psoriatics [108].

 Psychological stress may also enhance neurogenic inflammation in psoriatics. Harvima et al. evaluated involved and uninvolved skin from stressed and nonstressed patients through immunohistochemistry [109]. CGRP and vasoactive intestinal peptide nerve fibers were detected in the papillary dermis of the skin in stressed patients, whereas these nerve fibers were only weakly detected in nonstressed individuals. Moreover, concentrations of neuropeptide degrading enzymes (i.e., chymase) were decreased in stressed patients as compared with the nonstressed psoriatic controls.

 In summary, psoriasis is a multifactorial disease with a strong T-cell component. Psychological stress has been shown to trigger disease and exacerbate skin flares in some patients. Experimental psychological stressors have been shown to increase circulating levels of T cells, including T cells expressing the requisite proteins for skin homing (e.g., CLA). Second, the response of the HPA axis may be blunted in psoriasis patients with stress sensitivity. However, discordant results suggest that more studies are needed to determine the role of the HPA axis in psoriasis. Finally, psychological stress may enhance neurogenic inflammation in psoriatics.

## 5. Patient Treatment at the Intersection of Dermatology and Psychiatry

 Psychotropic medications (antipsychotics, lithium, antidepressants, and anticonvulsants) can lead to skin rash and skin allergy as well as severe skin reactions (Stevens-Johnson syndrome for treatment with anticonvulsants). On the other hand, adverse psychiatric effects to dermatological medications and treatments can include depression (e.g., isotretinoin and IFN*α* treatment) and psychosis (dapsone treatment). Locala has recently reviewed skin diseases caused or exacerbated by psychotropic medications as well as psychiatric adverse effects of dermatologic medications [110].

 The apparent psychophysiologic responses of many dermatoses may suggest that treatment programs structured at the dermatology/psychiatry interface may be useful for patient treatment, including programs that incorporate (1) psychotherapy, (2) biofeedback, (3) hypnosis, and (4) cognitive behavioral methods [111–113].

 Hypnosis is just one example of psychiatric treatment augmenting dermatological treatments for dermatoses. Shenefelt performed a MEDLINE search that covered the years 1966–1998 using search terms related to hypnosis and skin disease [111]. Results from MEDLINE showed that a wide range of dermatological disorders could be improved using hypnosis as an alternative or complementary therapy for skin disease treatment, including (1) atopic dermatitis, (2) psoriasis, (3) alopecia areata, (4) rosacea, (5) vitiligo, (6) hyperhidrosis, and (7) ichthyosis vulgaris [111]. Other psychiatric treatments may also benefit dermatology patients. For example, one study showed that patients who chose to participate in a cognitive behavioral therapy program reported reduced frequencies and numbers of psoriasis symptoms as long as 6 months after the program ended [114].

## 6. Summary and Conclusions

 The response to psychological stress is hypothesized to be an evolutionary adaptation for the fight-or-flight response. In contrast, for contemporary humans, activation of the HPA axis as a result of psychological stress can result in a number of undesirable physiological responses including the exacerbation of skin diseases. It has been shown that elements of the HPA axis as well as the sympathetic nervous system can modulate the innate and adaptive cutaneous immune responses, and a number of experiments have suggested that psychological stress can impact disease development and progression.

 Recent studies have shown that skin has its own HPA axis that may “fine tune” the response of the central HPA axis. The skin is especially sensitive to psychological stressors. Indeed, cooccurring psychiatric disorders are prevalent in patients with skin disorders. Both the innate and adaptive cutaneous immune responses are impacted by psychological stress as demonstrated in a number of experimental studies in both laboratory rodents and humans. Mouse models of contact hypersensitivity strongly suggest that the nature of the sensitizing agent, the dose of the contact sensitizing agent, and the timing of the stressor are all variables that are important for the ensuing immune response. Modulation of the cutaneous immune system by psychological stress most likely affects the course of skin diseases, including atopic dermatitis and psoriasis. Future investigations that explore the interconnections between psychological stress and the cutaneous innate and adaptive immune responses will enhance our understanding of skin immunology and immunological mediated skin diseases, provide unique insight into the mind and body connection, and may lead to new treatment programs that will improve patient care.
